# Device for Torsional Fatigue Strength Assessment Adapted for Pulsating Testing Machines

**DOI:** 10.3390/s22072667

**Published:** 2022-03-30

**Authors:** Viorel Goanta

**Affiliations:** Mechanical Engineering, Mechatronics and Robotics Department, Mechanical Engineering Faculty, “Gheorghe Asachi” Technical University of Iasi, 700050 Iasi, Romania; vgoanta@tuiasi.ro

**Keywords:** fatigue test, stress, strain, fatigue limit, device fatigue, calibration

## Abstract

The torsional fatigue test determines the fatigue limit for a certain asymmetry coefficient of the cycle. The assessment of fatigue tests is performed on specialized machines. There are two types of torsion testing machines: universal machines that have the torsion component and specialized machines only for torsion testing. Nevertheless, no matter which proposed option we choose, the purchase prices for these testing machines or the values spent for self-management are quite high. This paper presented a device used for torsion fatigue testing, adaptable to a universal pulsating testing machine, designed to determine the torsion fatigue limit for different materials. The built device is simple and reliable, and therefore inexpensive. By using this device, we can determine the limit of the torsional fatigue after any stress cycle and we can use the parameters obtained from the universal machine to which it was attached. The torque and twisting angle of the test specimen during the test can be determined by calculation. The paper also presented an experimental method for determining shear strains based on calibration experiment, using a specimen on which strain gauges were mounted. The values taken from this calibration experiment were compared with those obtained from the theoretical calculation.

## 1. Introduction

The main objective of this research was to present a device used for the torsional fatigue test. Most shafts that are subject to torsional loading and have a rotational motion are subject to variable shear stresses. For materials of which these components are manufactured, the fatigue strength must be determined, this being represented by the parameter τ_R_, named the limit of fatigue. This material characteristic is determined on the basis of torsional fatigue tests [1]. Experimental methods and, consequently, machines and devices, are known for determining the fatigue limit for tensile-compression loadings, bending, and torsion. The fatigue limit is determined for a certain R = τ_min_/τ_max_ asymmetry coefficient of the loading cycle. The options available are either specialized machines, built only for this purpose [2,3,4,5], or universal machines that also contain the torsion variant [6,7,8,9]. Devices used for torsional fatigue testing are, usually, independent, using actuators and sensors to determine the oscillatory motion [10,11,12,13,14]. Devices or sensors for measuring forces or torques can be adapted for machines built for tensile tests [15,16]. Both the testing machines (specialized or not) and the devices could also provide information about compound fatigue stress, which introduces into the specimen both tensile stress (normal stress) and torsional stress (shear stress) [17,18,19,20,21,22]. Whatever variant is used, it must be possible to change both the maximum stresses values and the asymmetry coefficient of the load cycle, R. For the specially built machines, the modification of R is more difficult and it is performed with the help of special devices. Both variants used for torsional fatigue testing have the disadvantage of being overly expensive. These special machines also have the following disadvantages: they should only be used for the torsion test, not all of them can have any stress cycle with different R, and the parameters taken for the determination of the fatigue limit are not very precise. Finally, we will need to be able to measure torque as a function of the twisting angle, based on which we can calculate τ_max_ and τ_min_. The problem solved by the device presented in this paper is the determination of the limit of torsional fatigue, τ_R_, using a simple construction device that can be attached to a universal pulsating test machine, from which data can be obtained accurately, with very good values of the parameters needed to determine the fatigue limit and the number of loading cycles. The presented device eliminates the disadvantages presented above to the universal testing machines with torsion components or to the special machines, due to the fact that it is built simply and reliably, and consequently is low-priced, being adaptable to a pulsating-type testing machine and therefore using parameters obtained from it, which are very precise. Torsional fatigue tests can be made and the limit to torsional fatigue can be determined after different R stress cycles.

## 2. Torsion Fatigue Device Configuration

**Figure 1:**
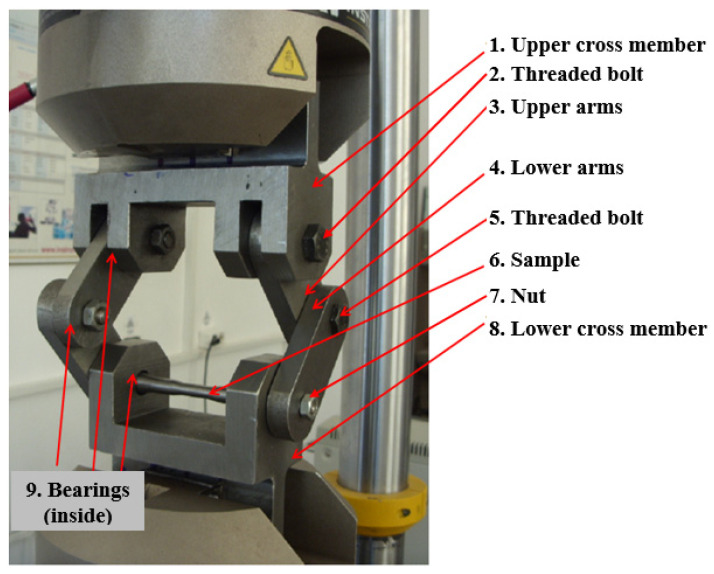
Torsion fatigue device—fixed in the pulsating testing machine.

This paper presents a device built to be able to test cylindrical samples for fatigue by torsion, therefore being able to determine the fatigue limit τ_R_. The device is designed in such a way that it can be attached to a universal pulsating type testing machine, Figure 1. Thus, the simplicity of the device is combined with the facilities offered by the universal pulsating testing machine, no longer requiring special testing machines. Under these conditions, all parameters regarding the load, displacement, frequency, and number of cycles were established on the basis of the software that drives the testing machine. On the other hand, the results were stored in the files within the process computer. The device is simple and adaptable to the universal pulsating test machine, containing two mechanisms, each with two articulated arms, the two mechanisms being placed anti-symmetrically in relation to the cross section of the sample and thus being able to insert two torques equal in size and in opposite directions at the ends of the sample.

**Figure 2:**
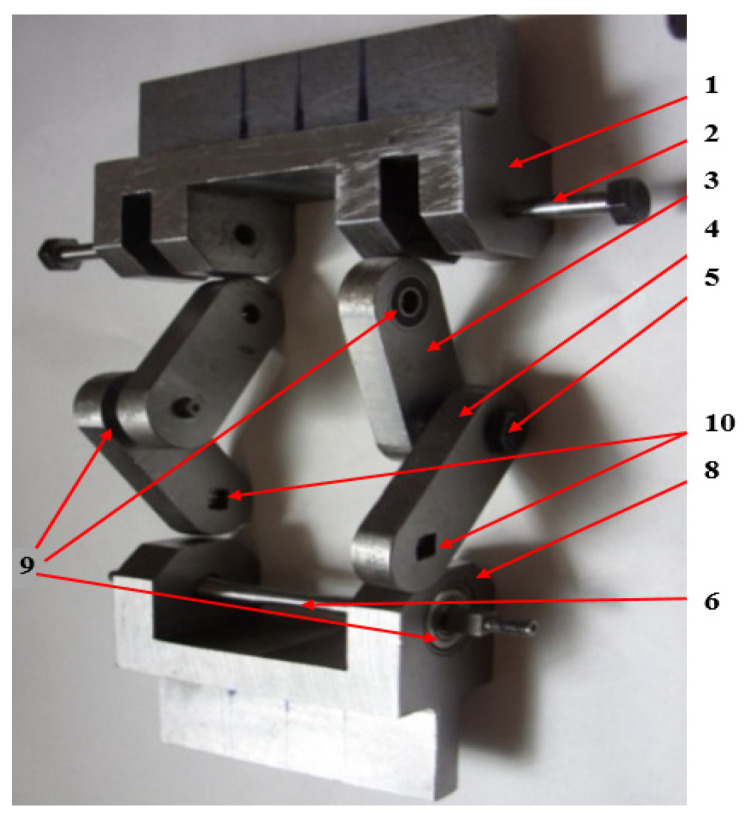
Device components and the sample.

**Figure 3:**
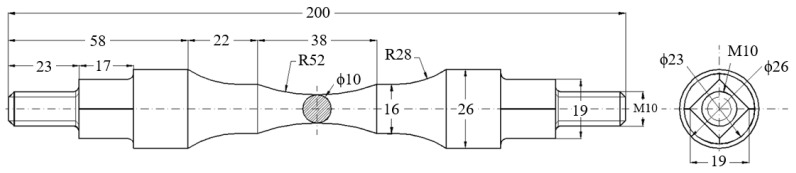
Geometric configuration and dimensions of the specimen.

The components of the device of Figure 2 are described below. At the top, the device contains an upper crossbar 1, which is fixed in the upper grip device of the testing machine, Figure 1. In the upper cross member, two arms, 3, (the upper ones) are fastened with the help of threaded bolts 2. In the upper arms, four radial bearings (not all being visible) are inserted, both at the top and at the bottom. The bearings are the radial ones, for bolts 2 and 5, which, in turn, constitute the cylindrical joints for arms 3 and 4. The arms 3 are placed anti-symmetrically, initially at an angle of 90° relative to each other. The lower arms 4 are articulated by the arms 3 by means of the threaded bolt 5. At the bottom part, the arms 4 have a clearance, 10, square in shape. This is provided for gripping the sample 6 which has the ends with the transversal section, also with square form, Figure 3.

The test sample 6 is hinged cylindrically in the lower crossbar 8 by means of two radial bearings, one of which is visible in Figure 2. The device is set in motion by the universal pulsating test machine by alternating motion of the upper crossbar 1 (for example), and holding the lower crossbar 8, which is fixed. By moving the upper crossbar 1 up and down, will move the arms 3 that will rotate, which will lead to a rotation of the arms 4. Being anti symmetrically arranged, the rotation of the two arms 3 will be carried out in the opposite direction, in relation to each other. The arms 4 will also rotate in the opposite direction, in relation to each other, thus creating equal but opposite two torsional moments at the ends of the specimen 6 by entraining it by the arms 4 through the areas of square section 10.

## 3. Determination of Torque Based on the Force and Displacement Provided by the Testing Machine

**Figure 4:**
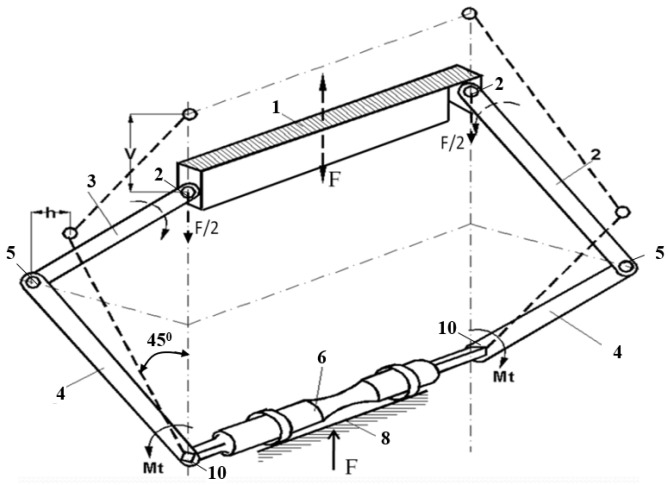
Device diagram—force F, displacement v, and moments M_t_.

In the following section, we determine the torque (torsion moment) applied to each of the ends of the specimen in order to calculate the maximum shear stress introduced into it. It is noted that from the test machine it is possible to accurately acquire values for the force F introduced by the machine and the displacement v of its piston. In Figure 4 it can be seen that the arms at the two ends of the specimen are placed anti-symmetrically, with the central joints determined by the bolts 5, on either side of the geometric axis of the specimen.

**Figure 5:**
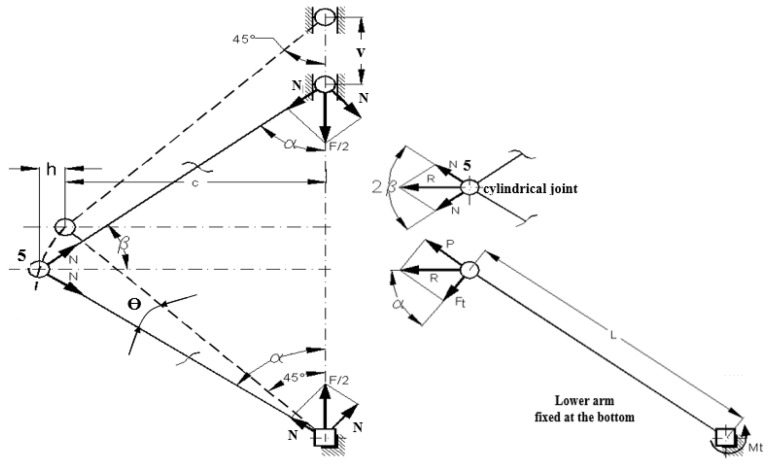
Representation of reaction forces in kinematic couplings and joints.

The distance between the two joints, measured perpendicular to the axis of the specimen, for the constructed device, is 88 mm. Under these conditions, during the vertical pulsating movement of the piston of the test machine, the two pairs of arms will perform opposite rotational movements, which allow the introduction at the ends of the specimen of equal and opposite torques. Thus, the requirement of a pure torsion test is satisfied. In order to determine the shear stress in the specimen, the torque moment Mt applied to the ends of the specimen must be evaluated as a function of the force F and the movement v recorded on the test machine. To determine a calculation formula in this regard, we represented the forces resulting in the joints of the device when the testing was carried out. Thus, in Figure 5, the way of designing and composing the reaction forces in the joints is shown. It should be noted that the initial equilibrium position and mounting of the specimen (force and moment are zero) is that shown with a dashed line in Figure 4, with an angle of 45° between the arms and vertical (direction of application) and, consequently, with the angle of 90° between arms 3 and 4, in the area of the central joint 5. When force is applied to the device from the test machine, the angle of the arms in relation to the vertical becomes α, Figure 5. The force, which is recorded in the data file, decomposes into arms 3 and 4 of the device into axial forces N and is calculated with the relation:

(1) N=F/2·cosα,

since the force F/2 is induced in a single pair of arms, see Figure 4.

In the central joint 5, the composition of the axial reaction forces N takes place, resulting in the force R, according to the relations:

(2) $$R^{2}=N^{2}+N^{2}+2\mathrm{NNcos}\;2\beta ,$$

On the other hand, the angle β is:

(3) β=π/2−α,

and it will result in:

(4) cos2β=cos2(π/2−α)=−cos2α,

Thus, the resulting force R in the central joint will be (from Equations (2) and (4)):

(5) $$R^{2}{=2N}^{2}{(1+\cos2\beta )=2N}^{2}\left(1-\cos2\alpha \right),$$

Given relations (1) and (5), the resulting force R will have:

(6) $$R=F\cdot \cos\alpha \sqrt{\frac{1-\cos2\alpha }{2}},$$

The resultant force R in the central joint 5 is decomposed on the lower arm, Figure 5, this being considered to be fixed at this moment, at the bottom, leading to the appearance of the force F_t_:

(7) $$F_{t}=R\cdot \cos\alpha =F\cdot \cos^{2}\alpha \sqrt{\frac{1-\cos2\alpha }{2}},$$

In relation to the gripping area of the test sample, the force F_t_ creates the torque $M_{t}$, which can be calculated with the relation:

(8) $$M_{t}{=F}_{t}\cdot L=F\cdot L\cdot \cos^{2}\alpha \sqrt{\frac{1-\cos2\alpha }{2}},$$

where L is the arm length visible in Figure 5.

**Figure 6:**
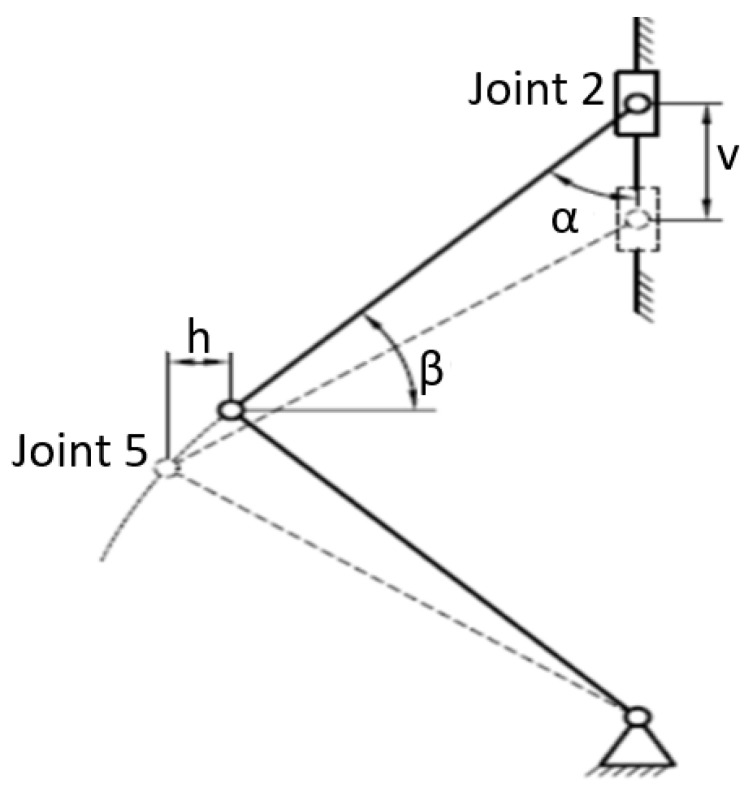
Representation of the displacement v and h.

We note that the torque is calculated based on the force F that is given by the test machine, the length L of the lower arm, which we can measure, and the angle α between the vertical axis of the device and the upper arms during operation. We cannot measure this angle, but we can determine it according to the vertical movement v of the upper crossbar, a value that is given by the test machine in the purchased data file. The kinematic diagram of the mechanism within the device of Figure 1 is presented in Figure 6.

**Figure 7:**
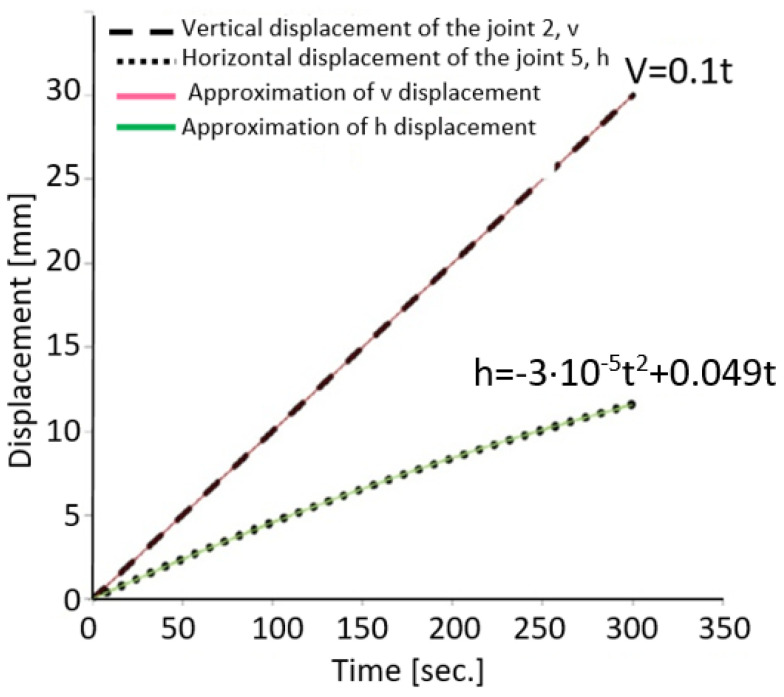
Displacement diagram.

With the help of specialized software, a simulation was performed based on a dynamic mechanism analysis, introducing geometry data and movement restrictions of the mechanism in question. Under these conditions, the program used provided the variations of the displacements of the points v, and h, in time, Figure 7. Eliminating the time from the relations presented in Figure 7, a relation of variation of the sizes v and h was obtained between them. Please note that this relationship is valid only for the dimensions of the mechanism built and shown in Figure 1. If the mechanism is constructed in the same way for the introduction of two equal torques and the opposite sign at the ends of the specimen, based only on the piston movement of the testing machine, in the software of the dynamic analysis of the mechanism type movement, the proper dimensions of the mechanism must be introduced, obtaining another variation between the sizes v and h.

From the relations for the two displacements, h and v, presented in Figure 7, the time is eliminated and relations be:

(9) $$h=-3\cdot 10^{-3}\cdot {\left(v\right)}^{2}+0.49\cdot v=f\left(v\right),$$

On the other hand, from the static mechanism—with the angle α = 45°—a relation is deduced between the length c, Figure 5, and the length L of the bar:

(10) $$c=\frac{L}{\sqrt{2}},$$

From the moving mechanism, Figure 5, the relationship is deduced:

(11) $$\sin\alpha =\frac{c+h}{L},$$

Given the relationships (9)–(11), the result is:

(12) $$\sin\alpha =\frac{\frac{L}{\sqrt{2}}+(-3\cdot 10^{-3}v^{2}+0.49v)}{L},$$

Thus, the angle α is given by the relation below, which is a function of the variable vertical displacement of the test machine piston.

(13) $$\alpha =\arcsin\left(\frac{1}{\sqrt{2}}-\frac{3\cdot 10^{-3}v^{2}-0.49v}{L}\right),$$

The angle α thus obtained is introduced in relation (8) by means of which the torque $M_{t}$ is calculated. Under these conditions, maximum shear stresses τ, calculated for the calibrated area of the sample, is given by:

(14) $$\tau =\frac{M_{t}}{W_{p}}=\frac{{F\cdot L\cdot \cos}^{2}\alpha \sqrt{\frac{1-\cos2\alpha }{2}}}{\frac{\pi d^{3}}{16}},$$

where α is calculated with relation (13) and $W_{p}$ is the moment of polar inertia which, for a specimen of circular section of diameter d, is calculated with the relation:

(15) $$W_{p}=\frac{\pi d^{3}}{16},$$

The angle Ɵ with which the test sample will rotate, Figure 5, is given by the relation:

Ɵ = α − 45,(16)

*Important note: Figure 5 shows that a force (P) appears on each of the arms 4, along the respective arms. The horizontal components of these forces, on each of the arms 4, create a moment of rotation, in the same direction, the direction of the resulting added moment being in the direction of the loading axis of the test machine. This moment of rotation, occurring due to the asymmetry of the device, will be taken over by the cross member of the test machine. Under these conditions, care must be taken to mount the device in the test machine so that this torque is set in the direction of tightening the piston nuts of the machine.*

## 4. Results Obtained When Loading Samples Using the Device

Table 1 shows the results that can be obtained for shear stresses, using the relation (14), under the conditions of using the data file for taking over the displacement v and the force F from the test machine, (here we considered L = 60 mm and d = 10 mm). Data for F and v in Table 1 were obtained by a static preliminary test, with the device initially mounted at 45°, and a steel sample was used. The force increased, with the sample mounted, and the values v (displacement of the test machine piston) and F (force) were recorded in the elastic range. The variation of the force in relation to the displacement takes into account the behavior of the material, respectively, the correlation to the test (static or fatigue) between F and v, considering that the fatigue request is also made, initially, in the elastic field.

As can be seen from relation (14), the shear stresses introduced in the calibrated zone, of diameter d of the specimen, depend on the force given by the test machine, the length L of the arms, measurable, and the vertical displacement of the testing machine piston, v. Table 1 shows the calculated values of shear stresses based on the calculation relationships mentioned above and on the F-v ratio established on a steel sample. The calculation for the shear strain, γ, is [23]:

(17) $$\gamma =\frac{\tau }{G}=\frac{M_{t}}{G\cdot \mathrm{Wp}}=\frac{16M_{t}}{G\cdot {\pi d}^{3}},$$

For G, the value of 82,000 MPa was considered, d being equal to 10 mm.

## 5. Calibration Experiment Using Samples with Strain Gauges Mounted

In order to verify the correctness of the mathematical calculation relations, an experimental determination was used based on the mounting of two strain gauges on a steel sample—the same material as the one used in determining the F-v dependence in Table 1.

**Figure 8:**
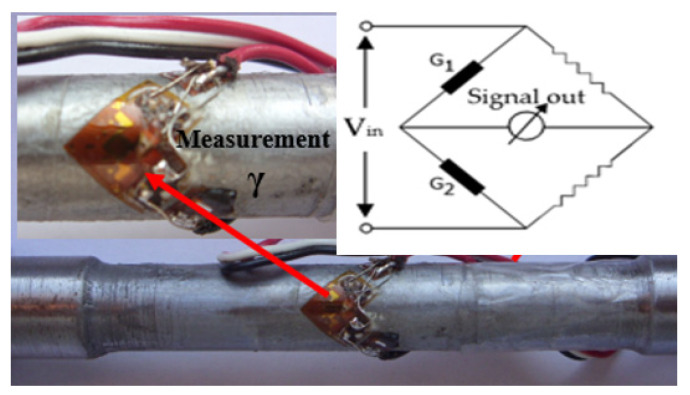
Measuring shear strain using tensometric transducers (two strain gauges).

**Figure 9:**
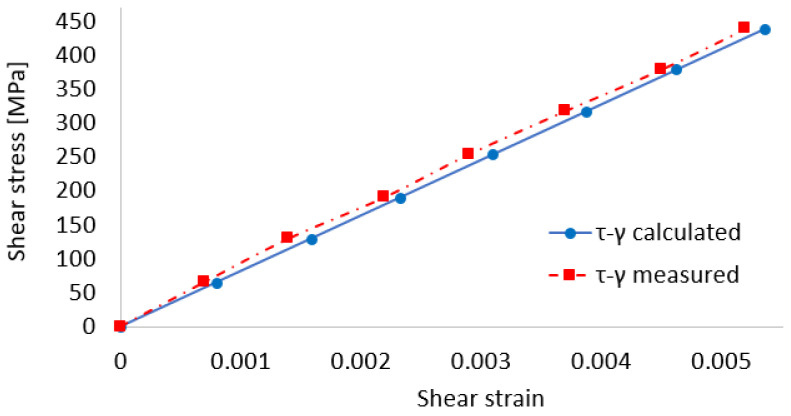
The shear stress versus shear strain variations, calculated and experimentally measured.

The two strain gauges were mounted on the sample, Figure 8, and connected in the Wheatstone half bridge. In these conditions, the shear strain will result according to the relation:

(18) γ=2ε,

Figure 9 shows the variation of shear stresses versus shear strains in the two situations: results from previous computational relationships and with the experimental determination of the shear strain. It is noted that the values resulting from the experimental determinations are slightly overestimated, but very close to the calculated values. Under these conditions, it can be stated that the relationships presented above can be used to determine the values of the following characteristics: torque and shear stress (maximum and minimum).

## 6. Conclusions

**Figure 10:**
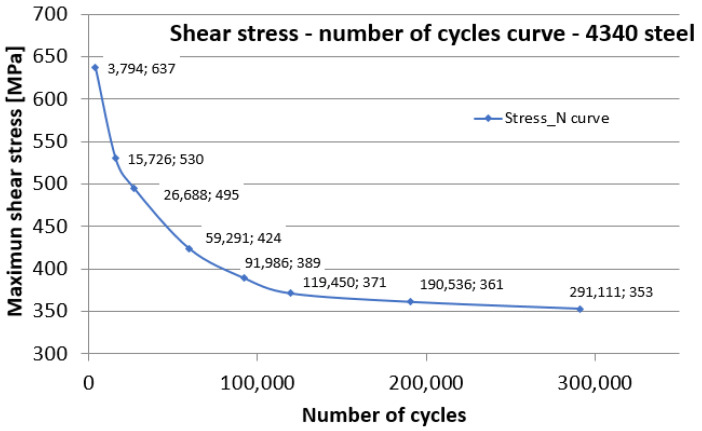
τ-N fatigue durability curve for 4340 steel.

Given the previous calculations, we have the necessary data to calculate the shear stress τ, and, after several tests, we can draw the Wöhler curve. Figure 10 shows the τ-N durability curve, obtained by torsion using the device presented in the paper, for steel 4340. For comparison, a durability curve is observed for the same steel in [24], for normal stresses, knowing that τ_R_ ≈ (0.6–0.7)·σ_R_. For a loading cycle, we calculated the maximum shear stress, which, together with the number of cycles to break, forms a point on the Wöhler diagram.

The value of the shear stress for which the specimen no longer breaks, after a sufficient number of stress cycles, is considered to be the limit to torsional fatigue, τ_R_. It is specified that the present device can achieve different values of the asymmetry coefficient R, based on the preload with an initial force, F_m_, and the fatigue load with an amplitude F_a_, around F_m_. In this sense, the device was mounted with arms 3 and 4, initially at an angle of 90 degrees, and, for an asymmetric cycle, statically, the upper crossbar was moved until the force Fm was reached, corresponding to a shear stress, τ_m_. Based on the previous calculations, for a certain maximum shear stress and a certain minimum shear stress, the amplitude of the test force F_a_ was determined. In this way, different values could be obtained for τ_min_ and τ_max_, respectively for the asymmetry coefficient of the stress cycle, R = τ_min_/τ_max_. Table 1 also shows the values for the torque Mt and the shear stress τ, calculated as a function of the force F and the displacement v of the piston of the test machine. Here, the sample was considered to have a diameter of 10 mm in the central area, Figure 3. Thus, with the help of the device shown in Figure 1 and Figure 2, a relatively high shear stress can be introduced into the sample so that it breaks after a lower number of load cycles. Under these conditions, it will be even more possible to introduce low stresses into samples that will withstand a larger number of cycles. The device presented in this paper, made as shown in Figure 1, can only be used for circular-section specimens and for materials that do not exceed a maximum shear stress of 750 MPa.

## Figures and Tables

**Table 1:** Results obtained for the determination of Mt based on F and v.

F	v	α	sin α	cos 2α	cos ^2^α	M_t_	τ
(N)	(mm)	(Degrees)				(N∙m)	(N/mm^2^)
0	0.0000	45.000	0.70711	0.0000000	0.500000	0.000	0.000
607	0.0098	45.006	0.70719	−0.0002257	0.499887	12.881	65.601
1202	0.0173	45.011	0.70725	−0.0003986	0.499801	25.495	129.846
1768	0.0232	45.015	0.70730	−0.0005349	0.499733	37.502	190.994
2352	0.0319	45.021	0.70737	−0.0007377	0.499631	49.879	254.030
2944	0.0412	45.027	0.70744	−0.0009522	0.499524	62.414	317.873
3512	0.0527	45.035	0.70754	−0.0012182	0.499391	74.449	379.164
4069	0.0603	45.040	0.70760	−0.0013919	0.499304	86.265	439.343
